# Photodynamic Therapy with 5-Aminolevulinic Acid Patch for the Treatment of Actinic Keratosis

**DOI:** 10.3390/jcm11113164

**Published:** 2022-06-02

**Authors:** Norbert Kiss, Klára Farkas, Giulio Tosti, Federico De Gado, Beata Bergler-Czop, Gilda Fazia, Antonella Tammaro, Carmen Cantisani

**Affiliations:** 1 Department of Dermatology, Venereology and Dermatooncology, Semmelweis University, 1085 Budapest, Hungary; kiss.norbert@med.semmelweis-univ.hu (N.K.); farkas.klara@phd.semmelweis.hu (K.F.); 2 Melanoma and Soft Tissue Sarcoma Division, IRCCS, European Institute of Oncology, 20141 Milan, Italy; giulio.tosti@gmail.com; 3 Department of Dermatology and Venereology, Policlinico Umberto I, Sapienza University of Rome, 00161 Rome, Italy; degadof@hotmail.com (F.D.G.); gilda0209@yahoo.it (G.F.); 4 Department of Dermatology, Medical University of Silesia, 40-055 Katowice, Poland; bettina2@tlen.pl; 5 NESMOS Dermatology Department, Sapienza University of Rome, 00189 Rome, Italy; antonella.tammaro@uniroma1.it

**Keywords:** 5-aminolevulinic acid patch, photodynamic therapy, PDT, daylight, treatment, actinic keratosis, actinic cheilitis, non-melanoma skin cancer

## Abstract

Photodynamic therapy (PDT) using 5-aminolevulinic acid (5-ALA) is an emerging treatment option in the care of actinic keratosis (AK). A self-adhesive 5-ALA patch was recently developed that allows a precise PDT procedure. Here, we review the current literature and report the findings of our case series that observed the outcomes and safety of 5-ALA patch PDT. Ten patients with a total of 40 AKs were treated with a single session of conventional or daylight PDT using 5-ALA patch at the Department of Dermatology and Venereology, Sapienza University of Rome or at the European Institute of Oncology, Milan, Italy. Complete response was observed in three patients, while partial response was seen in seven patients. Overall tolerability was good or excellent, with local adverse events observed in four patients. This is the first case series reported where the 5-ALA patch was applied using daylight PDT, and its efficacy and tolerability in the treatment of AK were demonstrated. In conclusion, the self-adhesive 5-ALA patch is a convenient application of PDT that provides a well-tolerated and effective treatment option with satisfactory cosmetic outcomes.

## 1. Introduction

Non-melanoma skin cancers (NMSC) are the most common types of malignancies in the fair-skinned population, showing an increasing incidence in recent decades [1]. Topical photodynamic therapy (PDT) can be a highly effective treatment for certain types of NMSC, including Bowen’s disease (BD), superficial basal cell carcinoma (sBCC) and selected cases of thin nodular basal cell carcinoma (nBCC) [2]. Importantly, PDT can also be effectively applied for the management of actinic keratosis (AK), a precancerous lesion of SCC. AK develops as a consequence of UV damage by chronic sun exposure, typically in sun-exposed skin areas of elderly individuals [3]. Current guidelines recommend treatment of AK lesions upon diagnosis in order to avoid progression to SCC [4]. Actinic cheilitis (AC), also referred to as AK of the lips, is a common form of AK. AC can also progress to SCC that harbors metastatic potential [5].

For PDT therapy, two prodrugs are available, 5-aminolevulinic acid (ALA) and its ester, methyl aminolevulinate (MAL). ALA and MAL penetrate the skin and are metabolized to protoporphyrin IX (PPIX), a precursor of heme which acts as the photosensitizer in PDT. PPIX accumulates in hyperproliferating tumor cells as they do not use oxidative phosphorylation and produce less heme. When excited by light of appropriate wavelengths, PPIX induces the generation of reactive oxygen species (ROS), mainly singlet oxygen, which damage tumor cells and ultimately lead to cell death [6].

ALA and MAL were found equally effective in the treatment of AK, BD, sBCC and nBCC in a recent retrospective study of 116 patients [7]. Typically, the application of ALA and MAL gels is followed by red light illumination, referred to as conventional PDT. Daylight PDT is also an emerging treatment modality, given that daylight also contains the necessary light spectrum to excite PPIX. While conventional PDT is an office-based procedure that requires three-hour occlusion of ALA or MAL before illumination, for daylight PDT, a short photosensitizer incubation period is sufficient. Moreover, while daylight PDT does not require equipment for illumination, it shows promising long-term efficacy [8]. Additionally, daylight PDT can be better tolerated, being generally less painful than conventional PDT [9]. The combination of daylight and conventional PDT with BP-200 ALA could further increase the efficacy of daylight PDT with higher clearance rates of AK lesions. However, a mild increase in the pain during the red light illumination was detected (visual analogue scale (VAS) 3.4 vs. 0.6) [10].

A self-adhesive, thin 5-ALA patch was developed to provide a more convenient application of PDT than when the active ingredient was formulated into a cream (Metvix^®^, Galderma, Lausanne, Switzerland), gel (Ameluz^®^, Biofrontera, Leverkusen, Germany) or alcoholic solution (Levulan^®^, DUSA Pharmaceuticals, Inc., Wilmington, MA, USA) [11]. Four hours following its application, a maximum of PPIX-specific fluorescence can be detected, with an insignificant elevation in 5-ALA plasma concentrations [12]. This product is now marketed as Alacare^®^ (Spirig Pharma AG, Egerkingen, Switzerland), a skin-colored dermal patch containing 8 mg ALA, and has been recently approved for the treatment of low-risk NMSC and mild to moderate AK. The patches measure 2 × 2 cm^2^ and contain a standardized amount of 2 mg ALA per cm^2^ [13].

In the present paper, we review the current literature and report our case series on the efficacy and safety outcomes of 5-ALA patch when used with conventional or daylight PDT.

## 2. Materials and Methods

### 2.1. Study Selection and Data Collection

The review of the current literature was constructed based on the Preferred Reporting Items for Systematic Reviews and Meta-Analyses (PRISMA) statement. We searched the PubMed, Scopus, Web of Science and Google Scholar databases. Studies were included where patients with AKs or AC were treated with PDT using the 5-ALA patch. The following keywords were used: “photodynamic therapy”, “patch”, “5-ALA patch”, “daylight”, “actinic keratosis” and “actinic cheilitis”. Number of patients and treated lesions, the utilized PDT methods, therapeutic response, the complete clearance rate, follow-up period and adverse events were collected.

### 2.2. Patients

Five female and five male patients were included in this case series managed at the Department of Dermatology and Venereology, Policlinico Umberto I, Sapienza University of Rome and at the European Institute of Oncology, Milan, Italy. Dermoscopic and clinical photos were collected from all examined lesions before and after treatment, with a 12-week follow-up time.

### 2.3. Inclusion Criteria

Adult patients with Fitzpatrick skin type I to III were included in this study with a clinical and dermoscopic diagnosis of AK confirmed by trained dermatologists before treatment. We examined lesions suitable for 5-ALA patch treatment. In order to pass our inclusion criteria, patients agreed to receive conventional or daylight PDT and to attend to the follow-up examinations in the 12 weeks after PTD.

### 2.4. Exclusion Criteria

Exclusion criteria included allergy to ALA or to the stimulating light sources, photosensitivity diseases such as porphyria, systemic lupus erythematosus, photosensitive dermatosis or patients who take photosensitizing drugs. In addition, patients who had skin diseases that might interfere with the treatment response evaluation were excluded. Patients who received any topical treatment within the previous 3 months or had chronic immunosuppression were also excluded.

### 2.5. Therapeutic Procedure

The lesions were treated with a single session of 5-ALA patch PDT. The 5-ALA patches were applied to the lesions for four hours, followed by the use of conventional or daylight PDT. During conventional PDT using light-emitting diode (LED) light sources, the lesions were illuminated with red light with a wavelength of 630 nm. The illumination time was 10 min with a total light dose of 37 J/cm^2^ at a light intensity of 61.7 mW/cm. Before daylight exposure, 5-ALA patches were applied to the lesions for 30 min. Daylight PDT was performed for two to three hours. The outcome was assessed clinically at 12 weeks after the PDT treatment.

## 3. Review of the Current Literature

### 3.1. Studies on the Use of Self-Adhesive 5-Aminolevulinic Acid Patch for Actinic Keratosis

A phase II, dose-finding study to investigate the efficacy and safety of 5-ALA patch PDT was carried out by Hauschild et al. The optimal application interval of the patch was assessed with illumination by fixed light dosage (37 J/cm^2^) and wavelength (630 nm). A total of 140 patients with 520 mild to moderate AK lesions completed the study. Eight weeks following the treatment, 86% of the AK lesions (74% of the patients) treated with 4-hour application of the patch achieved complete clearance, which is similar to the efficacy seen in conventional PDT. However, shorter application times of half, one or two hours showed inferior outcomes. Except for erythema, which was comparable to conventional PDT, the incidence of most local reactions including pain, desquamation and itching were lower when patch PDT was used [14]. The first two parallel-group phase III multicenter clinical trials on 5-ALA patch PDT were conducted on a total of 449 patients, with 103 patients included in AK 03 and 346 patients in AK 04. AK 03 compared the efficacy and safety of 5-ALA patch PDT with placebo–PDT by an observer-blinded superiority design. AK 04 was an open noninferiority study, where a comparison was made to cryotherapy. In the AK 03 and AK 04 studies, 12 weeks following treatment, clearance rates for 5-ALA patch PDT were 82% and 89%, while for placebo–PDT, clearance rates were 19% and 29%, respectively. In AK 04, using cryotherapy, 77% lesion clearance was achieved. Based on these data, 5-ALA patch PDT was proven superior to placebo–PDT and cryotherapy. Adverse events rated related to 5-ALA patch PDT therapy included transient discoloration of the skin in one case (AK 03), headache (similar incidence in the cryotherapy group), emotional distress and pyoderma (AK 04), with no serious adverse event reported related to study therapy. Mild and moderate degree local reactions, mainly itching due to 5-ALA patch application, were noted in 42% of the patients in AK04 and 34% in AK03, and no severe reaction was observed. Cooling of the illuminated lesions for relief was requested by 45% and 30% of the AK04 and AK03 patients, respectively, while illumination was stopped due to discomfort in only three patients [15]. In a 12-month follow-up study of 316 patients, Szeimies et al. concluded that a single course of 5-ALA patch PDT is superior to cryotherapy and placebo for the treatment of AK. Cosmetic outcomes proved to be better with patch PDT compared to cryotherapy. For the latter, significant hypopigmentation developed after treatment, while lesions treated with patch PDT showed normal pigmentation following clearance. Furthermore, the efficacy of the treatment was maintained for over a year, with up to half of the patch PDT-treated patients showing complete response. In contrast, conventional 5-ALA PDT or MAL-PDT often had to be repeated after 2–3 months due to incomplete clearance of the lesions, and curettage for crust removal was required before their application [13]. To date, only a sole case report of a 91-year-old male patient with moderate-grade AK with a facial localization has been published, where the 5-ALA patch was utilized for daylight PDT. A modified regimen was used, as 5-ALA patches were removed 30 min following application on individual AK lesions of the right eyebrow, right temporal region, nose and left cheek. After the removal of the patches, the patient was exposed to sunlight for two hours, with a non-reflecting sunscreen applied to all sun-exposed areas. The patient experienced no pain, and similar reactions developed as seen in conventional PDT. Three out of the four treated lesions resolved after seven weeks, leaving only mild erythema [16].

### 3.2. 5-Aminolevulinic Acid Patch for Actinic Cheilitis

A recent Austrian retrospective study reported evidence on the efficacy, tolerability and cosmetic outcomes of treatment with 5-ALA patch PDT in eleven patients with AC. After occlusion with 5-ALA patches for four hours, AC lesions were illuminated with narrowband red light for ten minutes with a cumulative dose of 37 J/cm^2^, without any pretreatment. A second 5-ALA patch PDT session was carried out after two weeks, although three patients with a total of five lesions were satisfied with the outcomes and refused a second treatment. Clinical assessments were carried out 3, 6, 9 and 12 months following treatment. Complete clinical response at the 3-month follow-up was achieved in 8 of 11 patients (72.7%) and 12 of 15 AC lesions (80.0%). Those patients, who had either a history of SCC or recurring AC, proved to be poor responders. At the time of the final 12-month follow-up, recurrence of only two lesions was observed. Thus, the complete clinical cure rate at one year after the use of 5-ALA patch PDT was 66.6% (10/15 lesions). These outcomes are superior to those reported for conventional MAL or ALA PDT. The most significant adverse event was pain during the treatment. For the first five patients, AC lesions were cooled during illumination for pain reduction. As the sixth patient noted very severe pain, local anesthesia was offered to all other patients, with notably reduced pain assessed by VAS. In all cases, after treatment, local phototoxic reactions developed, including swelling, blistering and erosions, which later resolved. Within one week following PDT treatment, labial herpes virus reactivation occurred in five patients, which could be well managed with valaciclovir. Cosmetic outcome of the treatment proved to be excellent in all cases. Even reduction in the perioral fine lines and increase in lip volume could be noted, with reduced lip dryness and roughness reported by the patients. Taken together, in this study, 5-ALA patch PDT was found to have substantial efficacy in the treatment of mild to moderate AC, with favorable cosmetic outcomes [5]. A further clinical trial extended this work by means of a prospective study on 21 patients. All patients received a single 10 min PDT session with red light at a dose of 37 J/cm^2^ four hours after the application of a 5-ALA patch. Patients received local anesthesia (mucosal injection of 1% mepivacaine) and a cold air blower was used on the lesions during PDT. Patients were clinically examined 2 and 7 days and 3, 6 and 12 months after PDT. Complete clinical response was achieved in 84% (16/19) of the patients, who completed the study. Local phototoxic reactions were mild to moderate, but oedema, blistering and erosions were severe in four patients. Moderate pain could be noted as the most significant adverse event. The mean VAS score was 3.4 (range 1–8) at the beginning, 4.5 (range 1–7) in the middle and 3.5 (range 1–7) at the end of the treatment period. Herpes virus reactivation occurred in three patients, which was successfully treated with valaciclovir. The cosmetic results were considered satisfactory (Table 1.) [17].

## 4. Results

Five female and five male patients with a mean age of 73.5 ± 8.37 years, ranging from 63–90 years, were treated with a single session of 5-ALA patch PDT at the Department of Dermatology and Venereology, Policlinico Umberto I, Sapienza University of Rome or at the European Institute of Oncology, Milan, Italy (Table 2).

**Figure 1:**
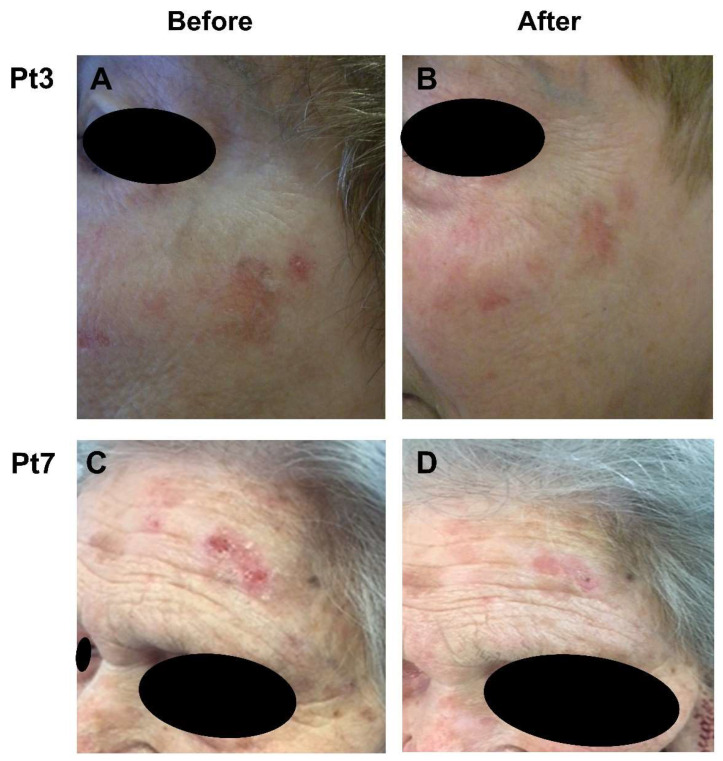
Comparing outcomes of conventional photodynamic therapy (PDT) using 630 nm 37 J/cm^2^ illumination (**A**,**B**) and daylight PDT (**C**,**D**) in patients with actinic keratosis (AK). Clinical photographs before (**A**,**C**) and 12 weeks after (**B**,**D**) treatment with a single application of 5-aminolevulinic acid patch. (**A**,**B**): Patient 3, left cheek, complete response. (**C**,**D**): Patient 7, left temple, partial response.

All included patients had Fitzpatrick skin type II or III and underwent treatment for a total of 40 AKs on the face (Figure 1). Three female and three male patients received conventional PDT, while two female and two male patients were treated with daylight PDT. Complete response (CR) was seen in three patients with 5.67 ± 0.47 AK lesions, all of whom initially received conventional PDT. However, in two of these patients, moderate erythema was noted, and in one patient, mild erythema was noted. Nevertheless, overall tolerability was still good in patients who achieved CR, with only mild pain. Partial response (PR) was observed in seven patients with 3.28 ± 0.95 skin lesions. Among these patients, three received conventional PDT, and the other four were administered daylight PDT. Considering the total numbers of treated AKs at baseline and at the end of follow-up, a 82.5% clearance rate was observed (33/40). Conventional PDT was used in six patients and 29 lesions were treated. Complete clearance was achieved in 26 lesions (89.65%). Using daylight PDT in four patients, 7 out of the 11 lesions showed complete clearance (63.6%) (Figure 1). Only one patient developed mild erythema, while mild pain was noted by three patients. Overall tolerability was good or excellent in patients who showed PR.

## 5. Discussion

PDT is an increasingly used treatment option in the field of dermato-oncology. The applications of ALA PDT are continually expanding with the development of novel devices, such as the 5-ALA patch PDT. Based on literature data and our own experiences, 5-ALA patch PDT is an effective modality for the management of both mild and moderate AK, and based on recent studies, also for AC. In accordance with the previous works, we applied 5-ALA patch PDT for AKs located on the face and scalp. Our results with a clearance rate of 82.5% overlap with the findings of the reviewed studies, which range between 75% and 86%.

Clinical trials on the efficacy of PDT with cream, gel and alcoholic solution 5-ALA and MAL formulations suggest that generally, the grade of AK lesions notably affects treatment outcomes. Interestingly, an in-depth analysis of the two completed phase III clinical trials investigating 5-ALA patch PDT revealed that there is no difference in efficacy or tolerability between mild and moderate AK lesions when treated with 5-ALA patch PDT. Additionally, neither the localization nor the size of AK lesions significantly influenced treatment efficacy. Comparing to other PDT procedures, the use of 5-ALA patch PDT does not require curettage or debulking before its application, not even for moderately thick hyperkeratotic AK lesions [13]. The assessment of AK lesion severity is not necessary, and often, a single session course proves sufficient for satisfactory outcomes [18]. In our patients undergoing 5-ALA patch PDT, all lesions improved, but, in some cases, had not completely resolved with a single treatment session. Later, in case of recurrency, or if certain lesions did not respond to the therapy, repeated treatments may be required to achieve higher clearance rates. When treating AC with a 5-ALA patch, no difference was found between the outcomes of single and double sessions. That study was limited by the small sample size, and the incomplete response may have been influenced by the immunosuppressed condition of the patients [5]. A detailed network meta-analysis was conducted to compare the efficacy of different treatments for AK of the head, including 5-ALA patch PDT, BF-200 ALA gel PDT, 3.75% and 5% imiquimod, 0.5% 5-fluorouracil (5-FU), diclofenac 3% in 2.5% hyaluronic acid, ingenol mebutate and cryotherapy. This work revealed that all of these treatment options were superior to placebo. While the authors concluded that BF-200 ALA gel PDT showed the highest complete patient clearance rate and also had the greatest probability to be the most efficient treatment, 5-ALA patch PDT was also among the most effective options [19]. However, it has been pointed out that this network meta-analysis has various major limitations, and its assumptions should be interpreted with caution [20].

Compared to the most commonly applied treatment modality, cryotherapy, 5-ALA PDT achieves better cosmetic outcomes and offers a higher ratio of complete lesion resolution for thin AKs located on the face and scalp [21]. However, in case of multiple lesions, several patches are required; thus, the high cost can be a limitation factor. While CR was achieved only in three patients in our case series, all the treated lesions showed substantial improvement. This suggests that repeated treatment may further improve efficacy.

To date, self-adhesive 5-ALA patch treatment using daylight PDT was reported only in one patient with AK [16]. In this case series, we applied this method in four patients and treated 11 lesions altogether. Partial responses were obtained with daylight PDT, but we applied it efficiently with minimal pain and no local skin reactions in all cases.

Following the PDT procedure, within hours, pain and local skin responses (erythema, edema, pustules, scaling) can occur. Patients should be informed about the recommended aftercare and the risk of the local skin reactions and their management [22]. In our patients, only mild pain and mild or moderate erythema were observed. The benefits of 5-ALA PDT include that it is well tolerated, with slight adverse effects. A study reported that daylight PDT could be used with minimal discomfort, while it was found to be as effective as conventional PDT for the treatment of thin and moderate-thickness AK [23]. Our results show similar outcomes. We found that patients treated with daylight PDT showed partial responses, but we applied it efficiently with minimal adverse reactions. By contrast, perceived pain from the conventional PDT is less predictable. No correlation was found between the Area and Severity Index (AKASI) and the PDT-associated pain [24].

### Advantages and Limitations

The advantages of the 5-ALA patch include its discreet and convenient application, that the patch provides light protection, and that no occlusive dressings, additional bandages or sunscreen are required. It allows accurate local dosing, and following incubation, one-step residue-free product removal is possible. Additionally, based on previous studies, 1 h incubation of the 5-ALA patch can be effective; however, the 4 h application showed the highest response rate [14]. Consequently, the use of 5-ALA patch PDT can be less time-consuming and it is less labor-intensive than other forms of PDT. Moreover, favorable cosmetic outcomes can be achieved, as most PDT-treated AK lesions show normal pigmentation one year after therapy, following a transient hyperpigmentation period [13]. It should be noted that the development of local phototoxic reactions correlates with treatment response, and they are not influenced by lesion grades [18]. For the 5-ALA patch PDT treatment of AC, exceptional cosmetic results were noted, while pain during the procedure seemed to be more severe and local anesthesia could be recommended [5,17]. The size of the patch-based approach can limit the complete clearance of the lesions. Large, widespread lesions cannot be covered completely by this application; thus, the borders of large lesions will not be treated. Few patients were involved in this study; therefore, no detailed statistical consequences could be shown. The fact that the response to AK was assigned by dermoscopic examination but not confirmed by histopathology can limit our study. The recurrence rate cannot be estimated with the relatively short follow-up period. The long-term outcomes need to be confirmed in the future, and a larger patient cohort should be examined.

To summarize, 5-ALA patch PDT is a novel modality for the treatment of AK and AC. It is a convenient application of PDT that offers an effective treatment option and provides satisfactory cosmetic outcomes. In addition, it is a well-tolerated treatment modality with mild or moderate local events and no systemic adverse events.

## Figures and Tables

**Table 1:** Data about actinic keratosis (AK) and actinic cheilitis (AC) treated with 5-aminolevulinic acid (5-ALA) patch photodynamic therapy (PDT) according to the included studies.

	First Author (Study Year)	No. of Pt	No. of Lesions	Type of PDT (5-ALA Patch Application Time)	Follow-Up	Complete Clearance	Adverse Event	Pain
Actinic keratosis	Hauschild et al. (2008) [14]	140	520	Conventional (0.5 h–1 h–2 h–4 h)	8 weeks	51%–72%–73%–86%	Mild to severe	Mild to severe
		34	130	Conventional (4 h)	8 weeks	86%	Mild to severe	Mild to severe
	Hauschild et al. (2009) [15]	69	384	Conventional (4 h)	12 weeks	82%	Mild or moderate	Mild or moderate 43%
		148	750	Conventional (4 h)	12 weeks	89%	Mild or moderate	Mild or moderate 35%
	Szeimies et al. (2010) [13]	174	NR	Conventional (NR)	12 months	79%	NR	NR
	Braathen et al. (2018) [16]	1	4	Daylight (0.5 h)	7 weeks	75%	Mild	Absent
Actinic cheilitis	Radakovic et al. (2017) [5]	11	15	Conventional (4 h) 2. PDT session in 8 lesions	12 months	66.6%	Moderate to severe	Mild to severe
	Radakovic et al. (2020) [17]	19	NR	Conventional (4 h)	12 months	84%	Mild, moderate, severe	Mild to moderate

Pt: patient; No.: number; PDT: photodynamic therapy; NR: not reported; 5-ALA: 5-aminolevulinic acid.

**Table 2:** Demographic data and clinical outcomes of our patients with actinic keratosis (AK) treated with a single application of 5-aminolevulinic acid patch.

Case No.	Sex	Age (Years)	Fitzpatrick Skin Type	Location	No. of Lesions	Light	Adverse Event	Pain	Overall Tolerability	Clinical Response at 12 Weeks
1	M	72	Type III	Forehead, cheeks	5	Conventional	Moderate erythema	Mild	Good	CR
2	F	76	Type II	Forehead, temple, nose	4	Conventional	Mild erythema	Absent	Excellent	PR
3	F	68	Type II	Forehead, cheeks	6	Conventional	Moderate erythema	Mild	Excellent	CR
4	M	69	Type III	Cheeks	6	Conventional	Mild erythema	Mild	Excellent	CR
5	F	71	Type II	Forehead, nose	4	Conventional	Absent	Absent	Excellent	PR
6	M	65	Type II	Right side of the scalp	4	Conventional	Absent	Absent	Excellent	PR
7	F	90	Type II	Left forehead	2	Daylight	Absent	Mild	Good	PR
8	F	78	Type III	Nose, left cheek, right temple	3	Daylight	Absent	Mild	Excellent	PR
9	M	83	Type II	Scalp	4	Daylight	Absent	Absent	Excellent	PR
10	M	63	Type II	Nose, left cheek	2	Daylight	Absent	Mild	Good	PR

CR: complete response; F: female; M: male; no.: number; PR: partial response.

## Data Availability

The data presented in this study are available on reasonable request from the corresponding author. The data are not publicly available due to ethical considerations.
